# Transmission Efficiency, Preference and Behavior of *Bemisia tabaci* MEAM1 and MED under the Influence of Tomato Chlorosis Virus

**DOI:** 10.3389/fpls.2017.02271

**Published:** 2018-01-17

**Authors:** Xiaobin Shi, Xin Tang, Xing Zhang, Deyong Zhang, Fan Li, Fei Yan, Youjun Zhang, Xuguo Zhou, Yong Liu

**Affiliations:** ^1^Key Laboratory of Pest Management of Horticultural Crop of Hunan Province, Hunan Plant Protection Institute, Hunan Academy of Agricultural Science, Changsha, China; ^2^College of Plant Protection, Hunan Agricultural University, Changsha, China; ^3^College of Life Sciences, University of Chinese Academy of Sciences, Beijing, China; ^4^College of Plant Protection, Yunnan Agricultural University, Kunming, China; ^5^Institute of Virus and Biotechnology, Zhejiang Academy of Agricultural Sciences, Hangzhou, China; ^6^Institute of Vegetables and Flowers, Chinese Academy of Agricultural Sciences, Beijing, China; ^7^Department of Entomology, University of Kentucky, Lexington, KY, United States

**Keywords:** tomato chlorosis virus, *Bemisia tabaci*, tomato, MEAM1 and MED, vector

## Abstract

Tomato chlorosis virus (ToCV, genus *Crinivirus*, family *Closteroviridae*) is an economically important virus in more than 20 countries. In China, ToCV was first detected in 2013 and has already spread throughout the country. ToCV is transmitted in a semi-persistent manner by the whitefly, *Bemisia tabaci*, but not seed. In the past two decades, the most invasive MEAM1 and MED have replaced the indigenous *B. tabaci* in China, and currently MED is the most dominant cryptic species. To better understand the prevalence of ToCV with their vectors, we tested the hypothesis that the rapid spread of ToCV in China is closely related to the dominance of MED. ToCV acquisition and accumulation rate following transmission was significantly higher by MED than MEAM1. In addition, ToCV persisted for more than 4 days in MED but only 2 days in MEAM1. Viruliferous MED preferred non-infected over virus-infected plants, although MED performed better on infected than on non-infected plants. Our combined results support the initial hypothesis that the rapid spread of ToCV is associated with the spread of *B. tabaci* MED in China.

## Introduction

Tomato chlorosis virus (genus *Crinivirus*, family *Closteroviridae*), an economically important virus worldwide (Orfanidou et al., 2014), was first identified in 1996 from tomato plants with symptoms of “yellow leaf disorder” (Wisler et al., 1998a,b). Over the past 20 years, ToCV has spread to more than 20 countries, such as North America, South America, Europe, the Middle East, Asia, and Africa (Wisler et al., 1998a; Accotto et al., 2001; Segev et al., 2004; Hirota et al., 2010; Arruabarrena et al., 2014; Fiallo-Olivé et al., 2011). ToCV can reduce tomato yield by 50–100% (Lozano et al., 2006; Velasco et al., 2008). ToCV is phloem-limited, and it causes interveinal chlorosis, leaf brittleness and limited necrotic flecking on tomato plants (Wisler et al., 1998b; Wintermantel and Wisler, 2006). ToCV is transmitted semi-persistently by whiteflies in the genera *Trialeurodes* and *Bemisia* (Wisler et al., 1998b; Navas-Castillo et al., 2000; Tzanetakis et al., 2013). *Bemisia tabaci* (Gennadius) (Hemiptera: Aleyrodidae) is a destructive pest worldwide. Previous studies showed that ToCV is transmitted more efficiently by *B. tabaci* MEAM1 or *Trialeurodes abutilonea* (Haldeman) than New World 1 (formerly biotype A) or *T. vaporariorum* (Wintermantel and Wisler, 2006)*. Bemisia tabaci* MED is also considered an effective vector of ToCV (Navas-Castillo et al., 2000; Orfanidou et al., 2016).

In mainland China, ToCV was first identified in 2012 associated with *Bemisia tabaci* MED and has subsequently spread throughout the country (Zhao et al., 2013; unpublished data). *Bemisia tabaci* MEAM1 and MED, formerly biotype B and Q (Polston et al., 2014), are the two most widely disseminated *B. tabaci* cryptic species worldwide. In China, *B. tabaci* MED has replaced MEAM1 in the past decade (Chu et al., 2010; Pan et al., 2012). We previously reported that tomato yellow leaf curl virus (TYLCV) is transmitted more efficiently by MED than MEAM1 and the dominance of MED has promoted the outbreak of TYLCV (Ning et al., 2015). In addition, *B. tabaci* MEAM1 and MED behaved differently on healthy and TYLCV-infected plants (Liu et al., 2013).

Based on the previous research with TYLCV, we hypothesize that the rapid spread of ToCV in China is associated with the dominance of MED. To test this hypothesis, we compared the acquisition, retention, and accumulation of ToCV following transmission by *B. tabaci* MEAM1 and MED. We also compared the preference and performance of *B. tabaci* MEAM1 and MED on healthy vs. ToCV-infected tomato plants.

## Materials and methods

### Tomato plants

The tomato plants (*Solanum lycopersicum* Mill. cv. Zhongza 9) were kept in whitefly-proof screen-cages in a greenhouse. To achieve systemic infection, plants at the three-true-leaf stage were injected with an infectious ToCV cDNA clone, and 0.5 ml of the infectious cDNA agro clone was injected into each plant (Zhao et al., 2016). The infection of was confirmed by the symptoms (chlorotic leaves) and by molecular detection at least 30 days post-inoculation using reverse transcription-polymerase chain reaction (RT-PCR). For RT-PCR analysis, RNA was extracted from the chlorotic leaves, and ToCV heat shock 70-like protein (HSP70h) was used to design the molecular primers (Table 1).

**Table 1:** Primers used in this study.

**Primer**	**Sequence (5′-3′)**	**Purpose**
ToCV-1F	AAACTGCCTGCATGAAAAGTCTC	ToCV RT-PCR
ToCV-1R	GGTTTGGATTTTGGTACTACATTCAGT	
ToCV-2F	ATGGAGAACAGT GCCGTTGC	ToCV qPCR
ToCV-2R	TTAGCAACCAGTTATCGATGC	
WF-F^a^	CTTGGTAACTCTTCTGTAGATGTGTGTT	*Bemisia tabaci* PCR
WF-R	CCTTCCCGCAGAAGAAATTTTGTTC	
Actin-F	CGCTGCCTCCACCTCATT	*Bemisia tabaci* reference genes
Actin-R	ACCGCAAGATTCCATACCC	
EF-1αF	TAGCCTTGTGCCAATTTCCG	
EF-1αR	CCTTCAGCATTACCGTCC	
Actin-F^b^	GGAAAAGCTTGCCTATGTGG	*Solanum lycopersicum* reference genes
Actin-R	CCTGCAGCTTCCATACCAAT	
UBI-F^b^	TCGTAAGGAGTGCCCTAATGCTGA	
UBI-R	CAATCGCCTCCAGCCTTGTTGTAA	

a *Primers WF originally reported by Chu et al. (2010) and Shi et al. (2013)*.

b *Primers Actin and UBI originally reported by Mascia et al. (2010)*.

### Insects

*Bemisia tabaci* MEAM1 (formely biotype B) and MED (formerly biotype Q) populations were established previously (Shi et al., 2013). The two populations were maintained separately on healthy (mock-inoculated) tomato plants in whitefly-proof screen-cages at 26 ± 2°C. The purity of the MEAM1 and MED populations was monitored every month based on a molecular marker, the mitochondrial cytochrome oxidase I gene, *mtCOI* (Table 1, Chu et al., 2010; Shi et al., 2013).

### Acquisition of ToCV

ToCV-infected plants were put in six screen-cages (eight ToCV-infected plants per screen-cage); three of them were used for MEAM1 acquisition and the other three for MED acquisition studies. Newly emerged MEAM1 and MED whiteflies were starved for 2 h before they were placed in clip-cages (20 adults of MEAM1 or MED /clip-cage). These clip-cages were attached to the fifth or sixth leaf from the top of ToCV-infected plants in the screen-cages (two or three clip-cages per plant). In each screen-cage, there were 10 clip-cages containing females and 10 clip-cages containing males. Male and female whiteflies were captured using transparent glass tubes with one open end (8 mm in diameter and 3 cm in height). Each whitefly was placed into one glass tube, and the open end of the glass tube was blocked with a piece of cotton. Sex was determined by observing the tail tip of whiteflies under a stereomiscroscope. After acquisition access period (AAP) of 0, 6, 12, 24, 48, 72, and 96 h, adults were collected randomly from the clip-cages on ToCV-infected leaves. Before collecting, plants were put on ice, clip cages were gently removed from leaves, and whiteflies were quickly collected using an aspirator. Different clip cages were used for each time point. All the cages were used only once. At each AAP, adults in three replicated screen-cages for each combination of cryptic species/sex were collected. The collected whiteflies were stored at −80°C for the subsequent ToCV detection.

### Retention of ToCV

**Figure 1:**
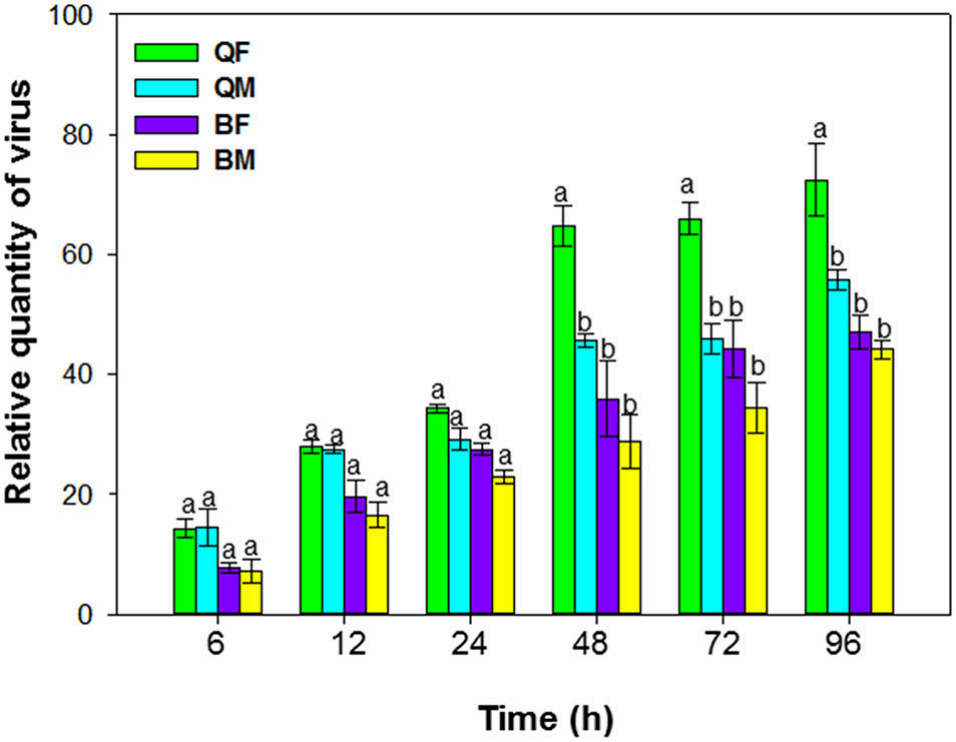
Acquisition of tomato chlorosis virus by *B. tabaci* MEAM1 and MED. QF, MED females; QM, MED males; BF, MEAM1 females; BM, MEAM1 males. Values are means ± SE. For each time, means with different letters are significantly different (*P* < 0.05; *N* = 3).

Retention of ToCV was assessed on cotton (*Gossypium hirsutum* L.), which is a host for MEAM1 and MED but not for the virus. Newly emerged MEAM1 and MED adults were starved for 2 h and then placed in separate screen-cages containing ToCV-infected tomato plants (five plants and approximately 400 whiteflies per screen-cage) for 48 h (our preliminary experiments showed that 48 h was optimal, Figure 1), which enabled the whiteflies to acquire their maximal viral loads. Viruliferous MEAM1 and MED whiteflies were then placed in clip-cages that were attached to cotton plants in screen-cages. There were six screen-cages, of which three were used for MEAM1 and three for MED retention studies. Each screen-cage contained eight cotton plants with 20 clip-cages, with 10 clip-cages containing females and 10 clip-cages containing males. After 0, 6, 12, 24, 48, 72, and 96 h on cotton plants, a total of 12 replicated clip-cages for each combination of cryptic species/sex in three replicated screen-cages were randomly collected (Supplementary Figure 1), and the whiteflies in these clip-cages were stored at −80°C for the subsequent ToCV detection.

### Accumulation of ToCV in tomato

After a 48-h AAP on ToCV-infected tomato leaves, newly emerged MEAM1 and MED whiteflies were collected and placed in clip-cages attached to healthy tomato plants at the three-true-leaf stage. Each plant had one clip-cage that contained a single female or a single male. Each of four treatments (a single MED female, a single MED male, a single MEAM1 female, and a single MEAM1 male) was represented by 10 clip-cages, with one clip-cage per plant, and each cage contained 10 tomato plants with 10 clip-cages, giving a total of 40 plants. After an IAP of 48 h, all of the whiteflies were removed, and the plants were then grown under insect-free and virus-free conditions in screen-cages. There were 16 screen-cages of four treatments (four cages per treatment), and four cages of the associated four treatments were sampled at one time point. After 30, 40, 50, and 60 d, the two youngest leaves of each plant were collected and stored at −80°C for detection of ToCV as described in the next section.

### Quantification of ToCV

RT-qPCR was used to detect and quantify ToCV after whitefly acquisition, retention, and ToCV accumulation following whitefly transmission experiments. ToCV-specific RT-qPCR primers, ToCV-2F and ToCV-2R, were designed according to the coat protein of ToCV. In addition, two pairs of reference genes were used for qPCR analysis, respectively, for whiteflies, *Actin-F* and *Actin-R, EF-1*α*F* and *EF-1*α*R*, and for tomato plants, *Actin-F* and *Actin-R, UBI-F* and *UBI-R* (Table 1). Total RNA was extracted from pooled whiteflies or each plant using the total RNA extraction kit (Tiangen Biotech, Beijing, China) and following the manufacturer's protocol. The first-strand cDNA was then synthesized with 1 μg of RNA using the PrimeScript® RT reagent kit (Takara Bio, Tokyo, Japan) with gDNA Eraser (Perfect Real Time, Takara Bio, Tokyo, Japan). The 25-μl reaction system contained 1 μl of cDNA, 12.5 μl of SYBR® Green PCR master mix (Tiangen Biotech, Beijing, China), 10.5 μl of RNase free ddH_2_O, and 0.5 μl of each primer. The relative quantities of ToCV were calculated based on the comparative cycle threshold 2^−ΔΔCt^ method (Livak and Schmittgen, 2001). Three biological replicates and four technical replicates per treatment were analyzed.

### Accumulation of ToCV in tomato following transmission by different numbers of MEAM1 and MED females

The methods described earlier to assess accumulation of ToCV in tomato were used to determine the effect of numbers of MEAM1 and MED females on transmission efficiency. Each of six treatments (a single MED female, 5 MED females, 10 MED females, a single MEAM1 female, 5 MEAM1 females, and 10 MEAM1 females) was represented by 10 clip-cages, with one clip-cage per plant, and each cage contained 10 tomato plants with 10 clip-cages. Each clip-cage contained 1, 5, or 10 females of MEAM1 or MED. After an IAP of 48 h, all of the whiteflies were removed, and the plants were then grown under insect-free and virus-free conditions in screen-cages. There were 24 screen-cages, of which 6 cages of the associated six treatments were sampled at one time point. After 30, 40, 50, and 60 d, the two youngest leaves of each plant were processed for detection of ToCV.

### Preference of whiteflies on ToCV-infected vs. healthy tomato plants

About 100 viruliferous or non-viruliferous MEAM1 or MED adults that had been starved for 2 h were collected by aspiration and released into each of 36 screen-cages (nine for non-viruliferous MEAM1, non-viruliferous MED, viruliferous MEAM1 and viruliferous MED, respectively). Each screen-cage contained one ToCV-infected tomato plant and one healthy tomato plant that were 40 cm apart. The whiteflies were released above the center of the two plants under dim light. After that all tests were conducted under a uniform incandescent light. The number of whiteflies on each infected and healthy plant was determined after 48 h. To prevent whiteflies from relocating during counting, the plants and associated whiteflies were covered with transparent plastic just before counting.

### Performance of whiteflies on ToCV-infected vs. healthy tomato plants

Newly emerged MEAM1 and MED females were collected and transferred to clip-cages attached to the third, fourth, fifth, and sixth leaf from the top of healthy and ToCV-infected tomato plants with chlorosis symptom on the lower leaves after injection of more than 30 days; there was one clip-cage per leaf and one female per clip cage. The eggs laid by each female were counted with a stereomicroscope (Leica, M205C) every 7 days, and then the clip-cages and whiteflies within were transferred to new plants. Every female was checked daily until its death, at which time longevity was calculated. There were four treatments: MED on healthy plants and on ToCV-infected plants, and MEAM1 on healthy plants and on ToCV-infected plants. Each treatment was represented by 30 clip-cages (females).

MEAM1 and MED nymph survivorship (the total number of emerged adults/the total number of eggs) and developmental time (from egg to adult) on healthy and ToCV-infected tomato plants were also determined. There were four treatments: MED on healthy plants and on ToCV-infected plants, and MEAM1 on healthy plants and on ToCV-infected plants. Each treatment was represented by 30 replicates. For each replicate, 20 newly emerged MEAM1 or MED adults (10 females and 10 males) were collected and placed in a clip-cage that was attached to a leaf of a tomato plant. The location of clip-cages was marked with a marker pen. After 24 h, the adults were removed. With a stereomicroscope (Leica, M205C), the leaves were examined and the eggs were counted; this required temporarily removing the clip-cages and then returning them to the original locations of the same leaves. From the 16th day, the leaves with clip-cages were examined twice daily (at 9:00 and 15:00). When newly emerged adults appeared in the clip-cages, they were collected every day (at 7:00 am in the morning) and the number was documented. The recording was continued until all the pupae had developed to adults (>35 days). The total number of emerged adults in each replicate was determined at the end of the experiment, and developmental times and survival rates were calculated.

### Data analysis

SPSS 22.0 (SPSS Inc., Chicago, IL, USA) was used for statistical analysis. Repeated-measures ANOVAs were used to compare acquisition, and retention of ToCV by *B. tabaci* MEAM1 and MED, and accumulation of ToCV in tomato following transmission by *B. tabaci* MEAM1 and MED. Repeated-measures ANOVAs were also used to compare the relative quantity of ToCV in tomato following transmission by *B. tabaci* MEAM1 and MED. The general linear model (GLM) was used to compare the preference and performance of MEAM1 and MED on healthy and ToCV-infected plants.

## Results

### Acquisition of ToCV by *B. tabaci* MEAM1 and MED

Acquisition of ToCV differed among MEAM1 and MED adults depending on the AAP [*F*_(3, 8)_ = 195.071, *P* < 0.001]. For AAPs of 6–24 h, the relative quantity of ToCV acquired did not differ among MEAM1 and MED adults (Figure 1). For both populations and sexes, the adults acquired maximal viral loads after a 48-h AAP, as the virus loads did not increase significantly after a 48-h AAP. From 48–96 h, the quantity of virus acquired was significantly greater for MED females than for MED males, MEAM1 females, or MEAM1 males (Figure 1).

### Retention of ToCV by *B. tabaci* MEAM1 and MED

**Figure 2:**
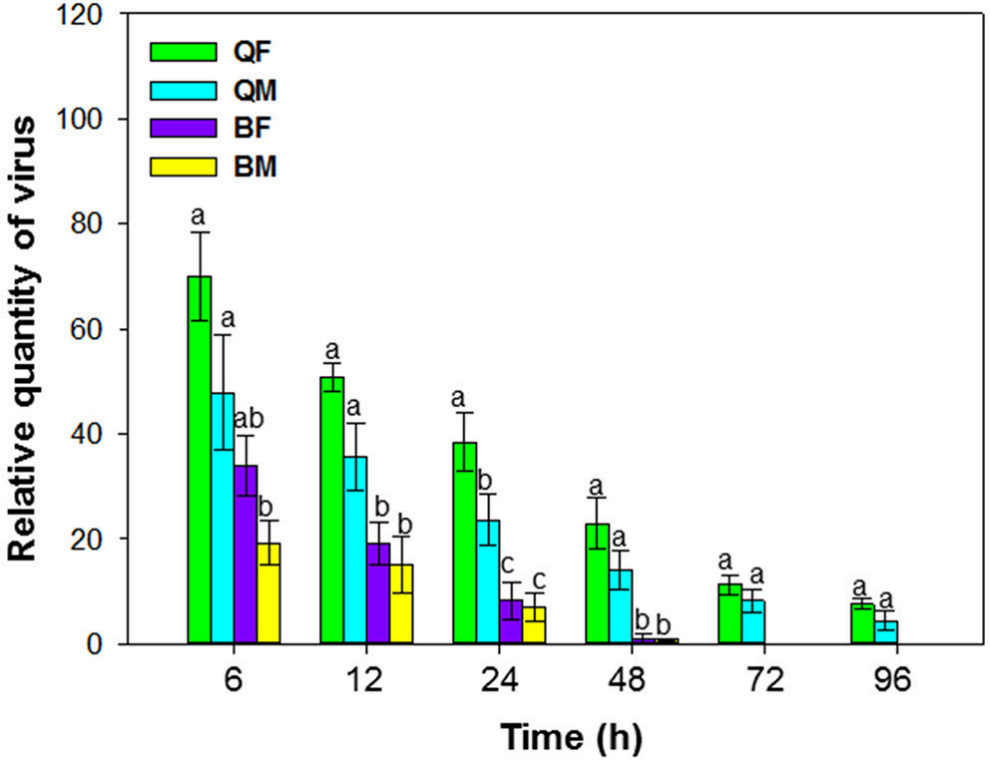
Retention of tomato chlorosis virus by *B. tabaci* MEAM1 and MED. QF, MED females; QM, MED males; BF, MEAM1 females; BM, MEAM1 males. Values are means ± SE. For each time, means with different letters are significantly different (*P* < 0.05; *N* = 3).

When viruliferous whiteflies were placed on cotton plants (hosts for the whiteflies but not for ToCV) for periods of 6, 12, 24, 48, 72, and 96 h, retention of ToCV differed among MEAM1 and MED females and differed with time [*F*_(3, 8)_ = 19.883, *P* < 0.001]. The virus titer of all the whiteflies decreased gradually with time (Figure 2). ToCV persisted for more than 96 h in MED females and males; retention was significantly higher in MED females than in MED males at 24 h and numerically higher than for males at other times, although results were not significantly different. In contrast, ToCV persisted for only 48 h in MEAM1 adults (Figure 2).

### Accumulation of ToCV in tomato following transmission by *B. tabaci* MEAM1 and MED

**Figure 3:**
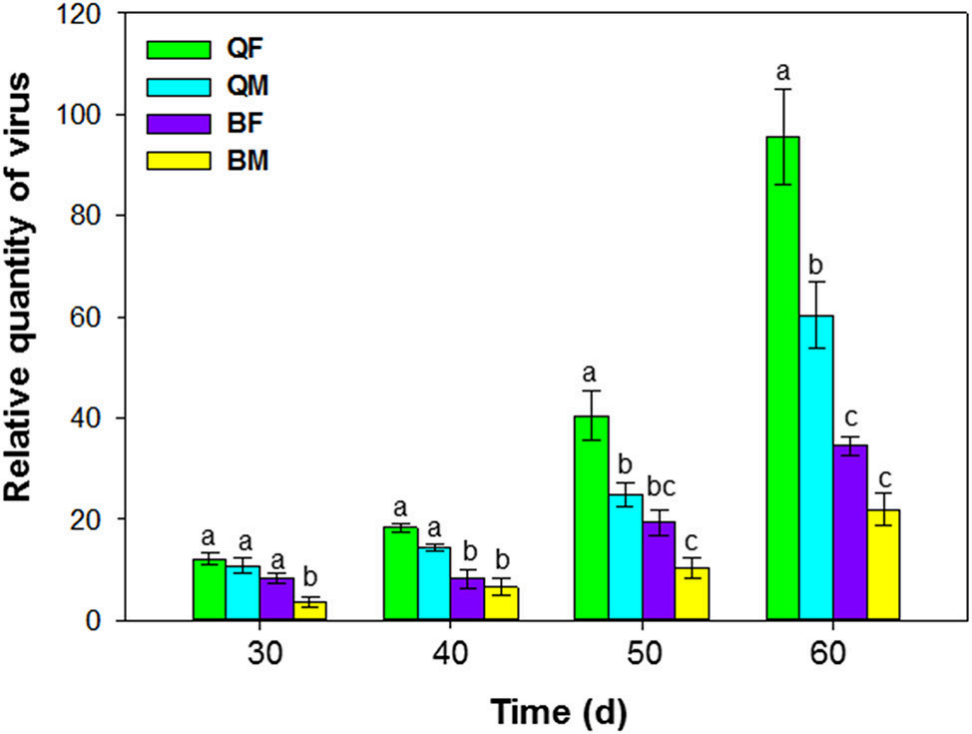
Accumulation of tomato chlorosis virus in tomato following transmission by *B. tabaci* MEAM1 and MED. QF, MED females; QM, MED males; BF, MEAM1 females; BM, MEAM1 males. The Y axis values refer to the relative quantity of virus detected in the two youngest leaves of each replicate plant. Values are means ± SE. For each time, means with different letters are significantly different (*P* < 0.05; *N* = 10).

Accumulation of ToCV in tomato following transmission by MEAM1 and MED adults differed at 30, 40, 50, and 60 d after inoculation [*F*_(3, 36)_ = 39.548, *P* < 0.001]. ToCV accumulation tended to be higher by the MED than the MEAM1 and by females than by males (Figure 3). At 50 and 60 d, ToCV accumulation was significantly higher by a single MED female than a single MED male, MEAM1 female, or MEAM1 male, respectively (Figure 3). At 60 d, accumulation of ToCV was significantly higher for a single MED male than a single MEAM1 female or male, respectively.

### Accumulation of ToCV in tomato following transmission by different numbers of *B. tabaci* MEAM1 and MED

**Figure 4:**
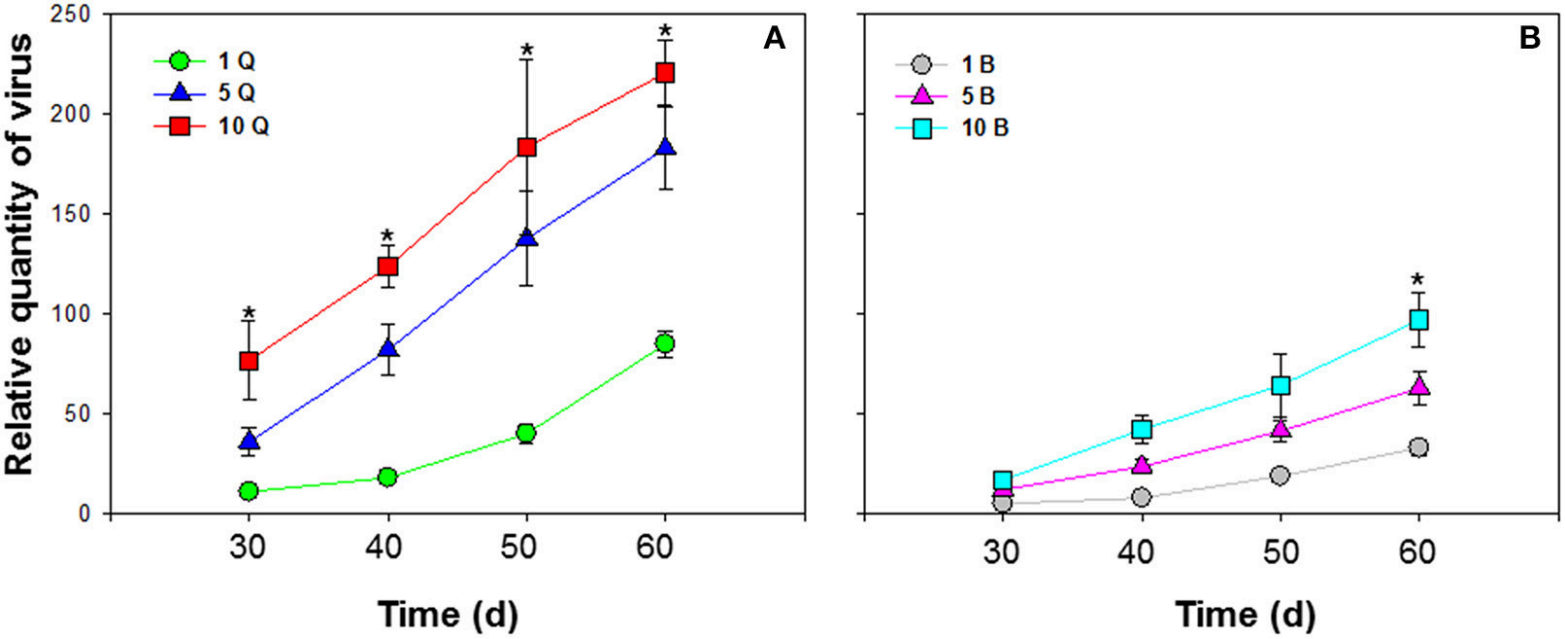
Accumulation of tomato chlorosis virus in tomato following transmission by different numbers of *B. tabaci* MEAM1 and MED females. **(A)** Accumulation of ToCV transmitted by different numbers of MED females. **(B)** Accumulation of ToCV transmitted by different numbers of MEAM1 females. 1 Q, 1 MED female; 5 Q, 5 MED females; 10 Q, 10 MED females; 1 B, 1 MEAM1 female; 5 B, 5 MEAM1 females; 10 B, 10 MEAM1 females. In the legend, 1, 5, and 10 indicate the number of MEAM1 or MED females added per clip-cage (one clip-cage per plant). The Y axis values refer to the relative quantity of virus detected in the two youngest leaves of each replicate plant. Values are means ± SE at *P* < 0.05; *N* = 10. An asterisk indicates a significant difference for MEAM1 (or MED) among number of 1, 5, or 10 at the same time point at *P* < 0.05.

Different numbers of *B. tabaci* MEAM1 and MED females were compared. ToCV was transmitted at the lowest levels by a single MEAM1 and MED whiteflies, and at intermediate levels by groups of 5 MEAM1 and MED whiteflies, and at the highest levels among the treatments by groups of 10 MEAM1 and MED whiteflies per plant [*F*_(2, 27)_ = 26.725 and *P* < 0.001 for MEAM1; *F*_(2, 27)_ = 52.861 and *P* < 0.001 for MED]. The increase in virus amount resulting from accumulation of ToCV with an increase in vector number was significantly greater for MED females than MEAM1 females [*F*_(1, 58)_ = 38.809, *P* < 0.001] (Figure 4).

### Preference of *B. tabaci* MEAM1 and MED on ToCV-infected vs. healthy tomato plants

**Figure 5:**
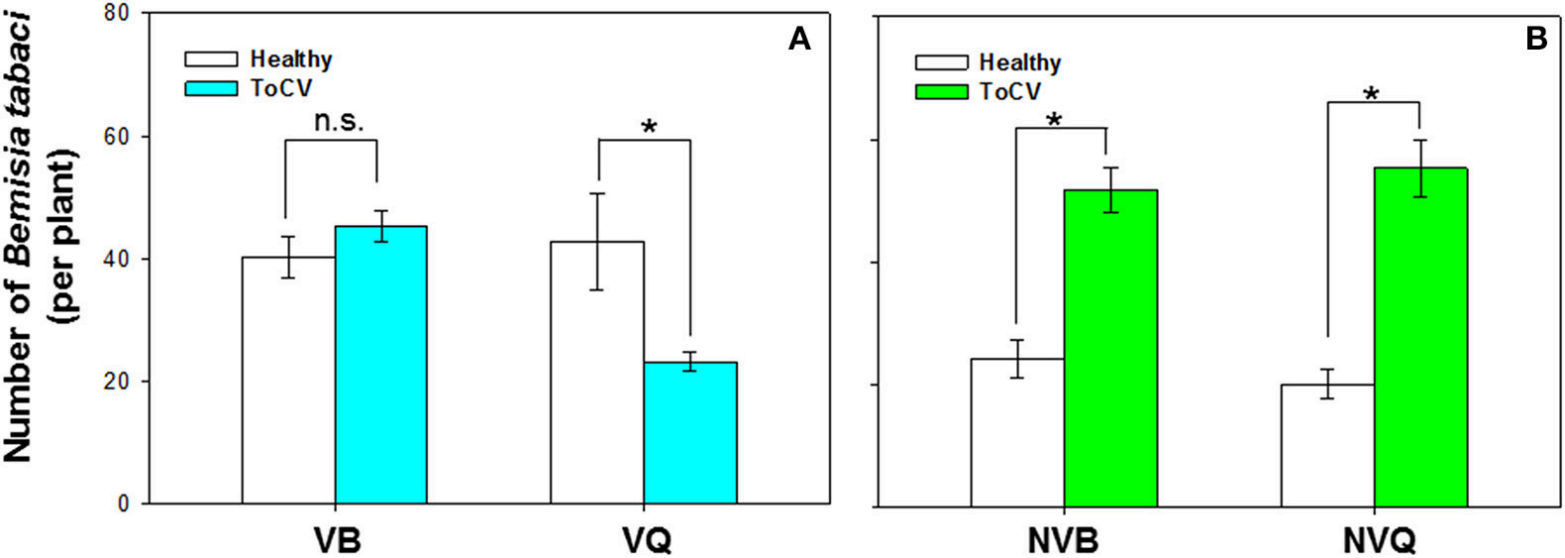
Preference of *B. tabaci* MEAM1 and MED for tomato chlorosis virus -infected vs. healthy tomato plants. **(A)** Preference of viruliferous MEAM1 and MED. **(B)** Preference of non-viruliferous MEAM1 and MED. VB, viruliferous MEAM1; VQ, viruliferous MED. NVB, non-viruliferous MEAM1; NVQ, non-viruliferous MED. Values are means ± SE. For each group of two means, an asterisk indicates a significant difference between the numbers on infected vs. healthy tomato plants (*P* < 0.05; *N* = 9).

*B. tabaci* preference was significantly affected by virus infection [*F*_(1, 71)_ = 17.372, *P* < 0.001], *B. tabaci* cryptic species [*F*_(1, 71)_ = 11.108, *P* < 0.001], and the interaction between virus infection and whitefly cryptic species [*F*_(1, 71)_ = 18.242, *P* < 0.001]. More viruliferous *B. tabaci* MED settled on ToCV-infected plants than on healthy plants, but similar numbers of viruliferous *B. tabaci* MEAM1 settled on ToCV-infected and healthy plants [MED: *F*_(1, 16)_ = 16.400, *P* = 0.026; MEAM1: *F*_(1, 16)_ = 0.885, *P* = 0.250; Figure 5A]. Non-viruliferous MEAM1 and MED were significantly more attracted to ToCV-infected than healthy plants [MED: *F*_(1, 16)_ = 4.863, *P* < 0.001; MEAM1: *F*_(1, 16)_ = 0.575, *P* < 0.001], and the number of non-viruliferous MEAM1 was numerically higher on control plants, but lower on ToCV-infected plants than the number of non-viruliferous MED (Figure 5B).

### Performance of non-viruliferous *B. tabaci* MEAM1 and MED on ToCV-infected vs. healthy tomato plants

**Figure 6:**
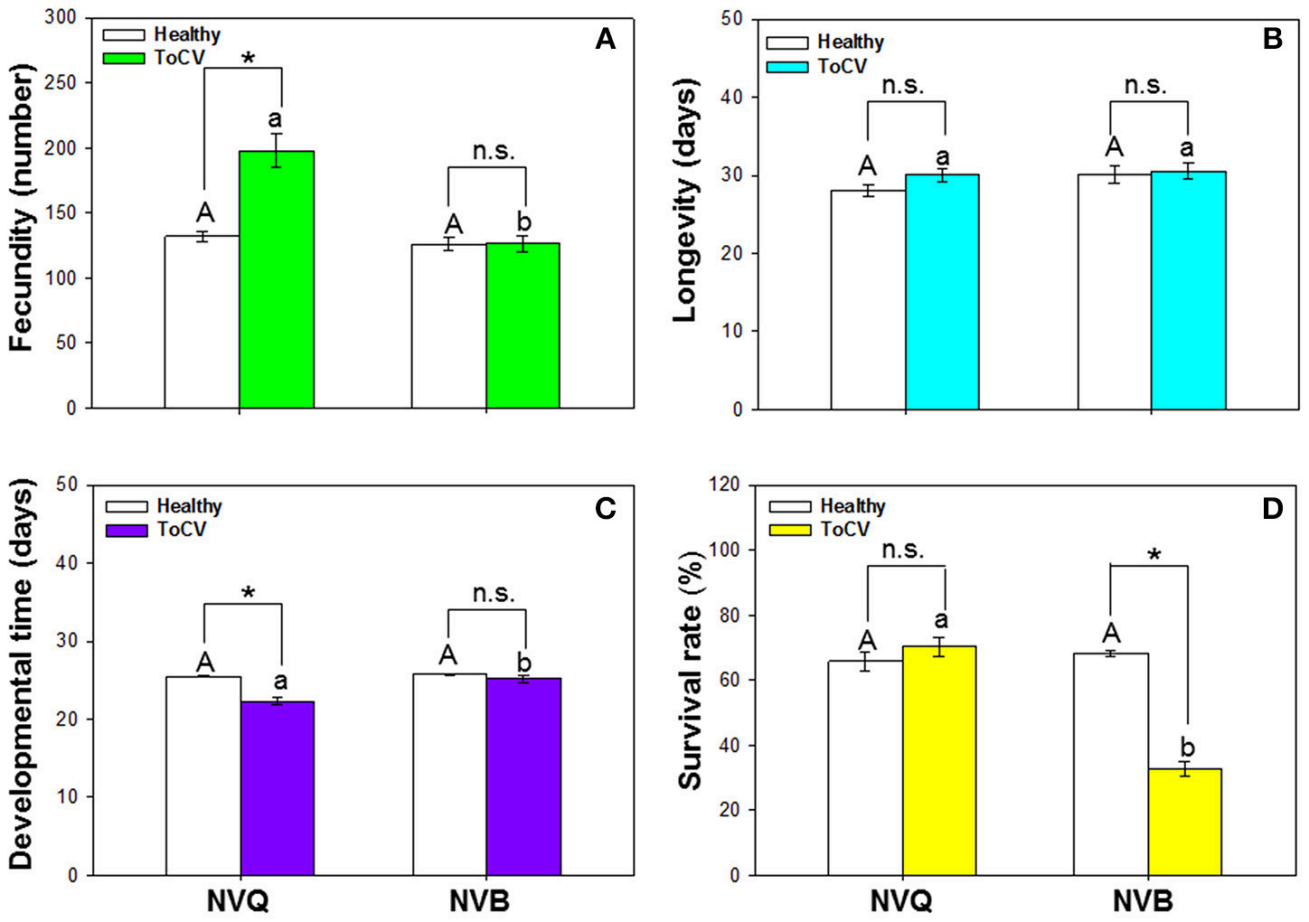
Performance of non-viruliferous *B. tabaci* MEAM1 and MED on tomato chlorosis virus -infected vs. healthy tomato plants. **(A)** Fecundity. **(B)** Longevity. **(C)** Developmental time. **(D)** Survival rate. NVB, non-viruliferous MEAM1; NVQ, non-viruliferous MED. Values are means ± SE. In each panel, means with different uppercase letters are significantly different for NVB vs. NVQ on healthy plants; means with different lowercase letters are significantly different for NVB vs. NVQ on infected plants (*P* < 0.05; *N* = 30); an asterisk indicates a significant difference for NVB (or NVQ) on healthy vs. infected plants; and n.s. indicates no significance for NVB (or NVQ) on healthy vs. infected plants (*P* < 0.05 for all comparisons).

*B. tabaci* fecundity was significantly affected by virus infection [*F*_(1, 101)_ = 21.415, *P* < 0.001, *N* = 101], *B. tabaci* cryptic species [*F*_(1, 101)_ = 29.082, *P* < 0.001, *N* = 101], and the interaction between virus infection and whitefly cryptic species [*F*_(1, 101)_ = 21.115, *P* < 0.001, *N* = 101]. Fecundity of MED was significantly higher on ToCV-infected plants than on healthy plants, while fecundity of MEAM1 was similar on ToCV-infected and healthy plants (Figure 6A). On ToCV-infected plants, fecundity was significantly higher for MED than MEAM1 (Figure 6A).

*Bemisia tabaci* longevity was not affected by virus infection [*F*_(1, 99)_ = 1.482, *P* = 0.226, *N* = 99], *B. tabaci* cryptic species [*F*_(1, 99)_ = 1.839, *P* = 0.178, *N* = 99], or the interaction between virus infection and *B. tabaci* cryptic species [*F*_(1, 99)_ = 0.668, *P* = 0.416, *N* = 99]. On ToCV-infected plants, longevity did not significantly differ between MEAM1 and MED (Figure 6B).

Developmental time of *B. tabaci* from egg to adult was significantly affected by virus infection [*F*_(1, 101)_ = 14.493, *P* < 0.001, *N* = 101], *B. tabaci* cryptic species [*F*_(1, 101)_ = 10.475, *P* = 0.002, *N* = 101], and the interaction between virus infection and *B. tabaci* cryptic species [*F*_(1, 101)_ = 6.671, *P* = 0.011, *N* = 101]. Developmental time of MED was significantly shorter on ToCV-infected plants than on healthy plants (Figure 6C). On ToCV-infected plants, developmental time was significantly shorter for MED than MEAM1 (Figure 6C).

Survival of *B. tabaci* was significantly affected by virus infection [*F*_(1, 96)_ = 37.217, *P* < 0.001, *N* = 96], *B. tabaci* cryptic species [*F*_(1, 96)_ = 47.033, *P* < 0.001, *N* = 96], and the interaction between virus infection and cryptic species [*F*_(1, 96)_ = 61.425, *P* < 0.001, *N* = 96]. Survival of MED was not affected by ToCV infection, while survival of MEAM1 was significantly lower on ToCV-infected plants than on healthy plants (Figure 6D).

## Discussion

ToCV is transmitted in a semi-persistent manner, and has spread throughout China since its first detection in 2012 (Zhao et al., 2013). We found that ToCV acquisition was significantly higher in MED females than in MED males or MEAM1 adults. Our previous finding showed that MED females acquired more of the persistently transmitted virus, TYLCV than MED males or MEAM1 adults (Ning et al., 2015). We also found that MED females fed better than MED males or MEAM1 adults on TYLCV-infected tomato plants (Ning et al., 2015). Therefore, the greater acquisition of ToCV in MED females might be attributed to the feeding behavior on ToCV-infected plants.

MEAM1 and MED differs in their ability to retain ToCV. ToCV persisted for more than 4 days in MED adults but for 2 days in MEAM1 adults. Some criniviruses persist for 1 day or less, while others can be retained for longer periods. This depends on both virus and vector. For example, ToCV persisted for only 1 days in New World 1 and *T. vaporariorum*, 2 days in MEAM1, up to 5 days in *T. abutilonea* (Wintermantel and Wisler, 2006), and at least 6 days in MED (Orfanidou et al., 2016). Retention of lettuce chlorosis virus (LCV) was less than 4 days, and cucurbit yellow stunting disorder virus (CYSDV) persisted in the vector for 9 days (Wisler et al., 1998a; Tzanetakis et al., 2013; Polston et al., 2014). In this study, we investigated and compared the retention of ToCV in MEAM1 and MED, respectively, under the same conditions. Our results showed that ToCV persisted in MEAM1 for 2 days, which is consistent with Wintermantel and Wisler (2006). The virus titer in MED remained at a high level at day-4. Based on the temporal change of virus titer in MED, we estimate that ToCV can persist in MED for at least 6 days, which is consistent with Orfanidou et al. (2016). Although the virus load in females was numerically higher than in males, the difference was not significant. The longer retention time in MED suggests that it may be a more effective ToCV vector.

When healthy plants were exposed to a single viruliferous whitefly adult (AAP = 48 h and IAP = 48), the ToCV titer in plants at 30-day post IAP was low and did not differ among the treatments, except when plants were exposed to single viruliferous MEAM1 males. In comparison to a large number of whiteflies, accumulation of viruses could be slowed down in individuals or smaller number of whiteflies. After 50 days, however, the virus titer was significantly higher in plants exposed to MED females than to MED males or MEAM1 adults. After 60 days, virus titer was still significantly higher in plants exposed to MED males than to MEAM1 adults.

In this study, transmission efficiency is also affected by the whitefly abundance. Accumulation of ToCV improved as the number of *B. tabaci* females per plant increased, and the increase in ToCV accumulation following transmission was significantly higher by MED females than MEAM1 females. A previous report showed that the efficiency of ToCV transmission by MED improved as the number of whitefly increased (Orfanidou et al., 2016). Here we determined the virus titer and found the similar trend. Additionally, ToCV accumulation rate by a single MEAM1 whiteflies was 14%, while it reached nearly 100% by 40 (Wintermantel and Wisler, 2006). The higher transmission efficiency and greater abundance of MED in the field may explain the rapid spread of ToCV throughout tomato producing regions in China.

MEAM1 and MED are the two most invasive whiteflies worldwide, and both of them are important virus vectors. During the last 20 years, MEAM1 has displaced the indigenous whiteflies in many countries, including the United States of America, Brazil and Australia (Costa and Brown, 1991; Perring, 2001; Lima et al., 2002; De Barro et al., 2006). Despite the worldwide dispersal of MEAM1, MED has started to displace MEAM1 in China, Japan and South Korea (Horowitz et al., 2003; Pascual and Callejas, 2004; Shatters et al., 2006; Pan et al., 2015). In China, insecticide application alters the competitive balance between MEAM1 and MED, as Chinese farmers predominantly rely on high-dose chemical treatments for pest and weed management (Xu et al., 2008). Pan et al. (2015) demonstrated that pesticide application is the driver of MED's displacement of MEAM1 throughout China, and consequently, it promoted the transmission of TYLCV.

While MED are closely linked with TYLCV prevalence in China and other Pacific Rim nations (Pan et al., 2012; Park et al., 2012), TYLCV transmission is primarily associated with MEAM1 in Israel (Gottlieb et al., 2010). In Spain and Costa Rica, MED is connected with ToCV infection (Navas-Castillo et al., 2000; Guevara-Coto et al., 2011); while, in Brazil, ToCV transmission is associated with MEAM1 (Barbosa et al., 2011). Both MEAM1 and MED are capable vectors of ToCV. There may be other factors causing rapid spread of ToCV in China and other parts of the world. In China, MED has developed a greater resistance to pesticides than MEAM1 (Pan et al., 2015). The heterogeneity of arable land and agricultural practices, especially pesticide application, are likely to contribute to the epidemic of ToCV.

In this study, viruliferous MED preferred to settle on non-infected plants rather than on ToCV-infected plants but viruliferous MEAM1 showed no preference. Nonviruliferous whiteflies of both MEAM1 and MED preferred ToCV infected plants over healthy plants. This behavior of whiteflies should promote ToCV transmission. Other studies have shown that persistently transmitted viruses can induce changes in the plant host or vector that enhance virus transmission. *Rhopalosiphum padi* aphids carrying barley yellow dwarf virus (BYDV), for example, prefer non-infected wheat plants, while aphids without BYDV prefer BYDV-infected plants (Ingwell et al., 2012). *B. tabaci* MED carrying TYLCV also preferred non-infected plants to infected plants while *B. tabaci* MED without TYLCV showed no preference for infected or non-infected plants (Fang et al., 2013). ToCV is a semi-persistent virus that is maintained in its whitefly vector for only a few days. Previous results also showed that viruliferous MEAM1 showed no preference between ToCV-infected and healthy plants (Fereres et al., 2016). Here, we found the same phenomenon that viruliferous MEAM1 showed no preference between ToCV-infected and healthy plants. Fereres et al. (2016) found that nonviruliferous MEAM1 preferred healthy plants, while we found that nonviruliferous MEAM1 were attracted to ToCV-infected plants. Difference in experimental procedures may cause this discrepancy.

When *B. tabaci* MED fed on ToCV-infected plants, its fecundity was increased and developmental time was decreased in comparison to ingesting healthy plants. This suggests that ToCV infection will increase MED abundance. Combined with the previous result we found that an increase in MED abundance substantially increases ToCV transmission, and this finding may help to explain why ToCV has spread so rapidly in China. The survival rate of MEAM1 but not MED was decreased by feeding on ToCV-infected tomato plants. Several factors can influence the survival of whiteflies, including environmental conditions, physiological conditions of the host plants and natural enemies. In this study, the environmental conditions were the same, and the physiological conditions of host plants induced by ToCV infection may be a major factor. Previous results showed that TYLCV infection of host plants increased MED growth and development but decreased MEAM1 growth and development (Pan et al., 2012). We also previously found that feeding by viruliferous MED but not by viruliferous MEAM1 can reduce the JA signaling pathway of plants to facilitate TYLCV transmission (Shi et al., 2014). ToCV infection may induce plant defense that has negative effects on MEAM1 survival, but this needs further study. Kaur et al. (2017) found a number of pathways with differential expression in response to ToCV infection. It is likely that differentially regulated pathways contribute to whitefly acquisition, retention, and ToCV accumulation. Future research can follow up on the interactions between ToCV and *B. tabaci* through essential biological pathways, including metabolism, signal transduction, and catabolism.

## Author contributions

XS and XT contributed equally to this work. XS, XGZ, and YL designed the experiments, XS and XT carried out all experimental work, XS, XT, and XGZ performed data analysis and wrote the manuscript, XZ, DZ, FL, FY, and YZ contributed to the preparation of experiment materials. All authors give final approval for publication.

### Conflict of interest statement

The authors declare that the research was conducted in the absence of any commercial or financial relationships that could be construed as a potential conflict of interest.

## Supplementary material

Supplementary Figure 1 Sample collection in retention of tomato chlorosis virus. There were seven time periods: 0, 6, 12, 24, 48, 72, and 96 h. At each time point, 12 clip-cages with 3 clip-cages of MEAM1 female, 3 cages of MEAM1 male, 3 cages of MED female, and 3 cages of MED male were collected. A total of 84 clip-cages were collected in this experiment.
