# Supplementary figures and images for: Transmission Efficiency, Preference and Behavior of Bemisia tabaci MEAM1 and MED under the Influence of Tomato Chlorosis Virus
Source: Front Plant Sci. 2018 Jan 17;8:2271. doi: 10.3389/fpls.2017.02271 (PMC5776130; doi:10.3389/fpls.2017.02271)

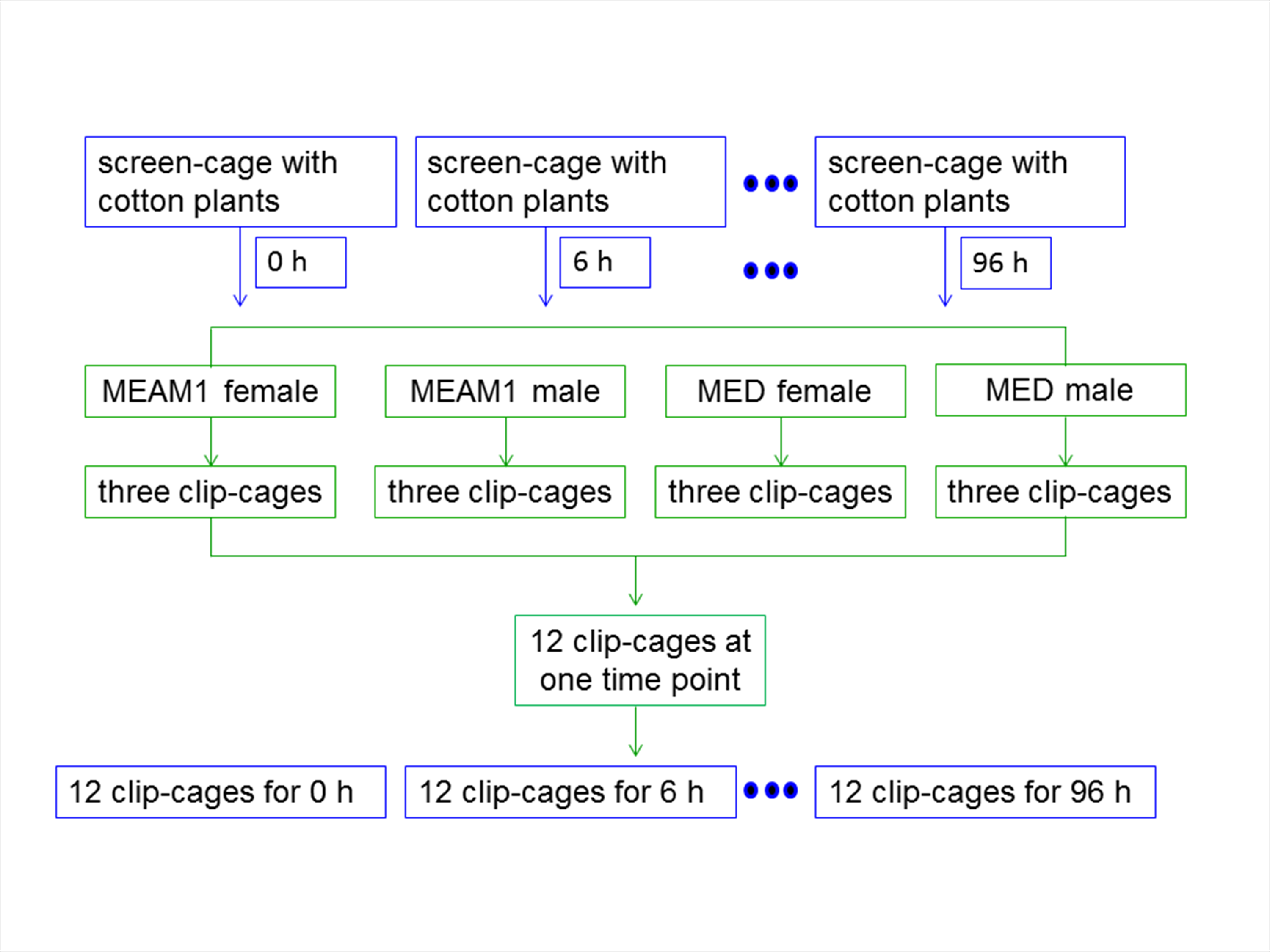

Supplement: Supplementary Figure 1 — Sample collection in retention of tomato chlorosis virus. There were seven time periods: 0, 6, 12, 24, 48, 72, and 96 h. At each time point, 12 clip-cages with 3 clip-cages of MEAM1 female, 3 cages of MEAM1 male, 3 cages of MED female, and 3 cages of MED male were collected. A total of 84 clip-cages were collected in this experiment. [file Image1.TIF]
